# Discovery of variant infectious salmon anaemia virus (ISAV) of European genotype in British Columbia, Canada

**DOI:** 10.1186/s12985-015-0459-1

**Published:** 2016-01-06

**Authors:** Molly JT Kibenge, Tokinori Iwamoto, Yingwei Wang, Alexandra Morton, Richard Routledge, Frederick SB Kibenge

**Affiliations:** Department of Pathology and Microbiology, Atlantic Veterinary College, University of Prince Edward Island, 550 University Ave., Charlottetown, P.E.I. C1A 4P3 Canada; Department of Computer Science, University of Prince Edward Island, 550 University Ave., Charlottetown, P.E.I. C1A 4P3 Canada; Raincoast Research Society, Box 399, 390 1st Street, Sointula, BC V0N 3E0 Canada; Department of Statistics and Actuarial Science, Simon Fraser University, 8888 University Drive, Burnaby, B.C. V5A 1S6 Canada; Current address: Diagnostic Services Unit, Atlantic Veterinary College, University of Prince Edward Island, 550 University Ave., Charlottetown, P.E.I. C1A 4P3 Canada

**Keywords:** Infectious salmon anaemia virus, ISAV, ISAV variant, European genotype

## Abstract

**Background:**

Infectious salmon anaemia (ISA) virus (ISAV) belongs to the genus *Isavirus*, family *Orthomyxoviridae*. ISAV occurs in two basic genotypes, North American and European. The European genotype is more widespread and shows greater genetic variation and greater virulence variation than the North American genotype. To date, all of the ISAV isolates from the clinical disease, ISA, have had deletions in the highly polymorphic region (HPR) on ISAV segment 6 (ISAV-HPRΔ) relative to ISAV-HPR0, named numerically from ISAV-HPR1 to over ISAV-HPR30. ISA outbreaks have only been reported in farmed Atlantic salmon, although ISAV has been detected by RT-PCR in wild fish. It is recognized that asymptomatically ISAV-infected fish exist. There is no universally accepted ISAV RT-qPCR TaqMan® assay. Most diagnostic laboratories use the primer-probe set targeting a 104 bp-fragment on ISAV segment 8. Some laboratories and researchers have found a primer-probe set targeting ISAV segment 7 to be more sensitive. Other researchers have published different ISAV segment 8 primer-probe sets that are highly sensitive.

**Methods:**

In this study, we tested 1,106 fish tissue samples collected from (i) market-bought farmed salmonids and (ii) wild salmon from throughout British Columbia (BC), Canada, for ISAV using real time RT-qPCR targeting segment 8 and/or conventional RT-PCR with segment 8 primers and segment 6 HPR primers, and by virus isolation attempts using Salmon head kidney (SHK-1 and ASK-2) cell line monolayers. The sequences from the conventional PCR products were compared by multiple alignment and phylogenetic analyses.

**Results:**

Seventy-nine samples were “non-negative” with at least one of these tests in one or more replicates. The ISAV segment 6 HPR sequences from the PCR products matched ISAV variants, HPR5 on 29 samples, one sample had both HPR5 and HPR7b and one matched HPR0. All sequences were of European genotype. In addition, alignment of sequences of the conventional PCR product segment 8 showed they had a single nucleotide mutation in the region of the probe sequence and a 9-nucleotide overlap with the reverse primer sequence of the real time RT-qPCR assay. None of the classical ISAV segment 8 sequences in the GenBank have this mutation in the probe-binding site of the assay, suggesting the presence of a novel ISAV variant in BC. A phylogenetic tree of these sequences showed that some ISAV sequences diverted early from the classical European genotype sequences, while others have evolved separately. All virus isolation attempts on the samples were negative, and thus the samples were considered “negative” in terms of the threshold trigger set for Canadian federal regulatory action; i.e., successful virus isolation in cell culture.

**Conclusions:**

This is the first published report of the detection of ISAV sequences in fish from British Columbia, Canada. The sequences detected, both of ISAV-HPRΔ and ISAV-HPR0 are of European genotype. These sequences are different from the classical ISAV segment 8 sequences, and this difference suggests the presence of a new ISAV variant of European genotype in BC. Our results further suggest that ISAV-HPRΔ strains can be present without clinical disease in farmed fish and without being detected by virus isolation using fish cell lines.

**Electronic supplementary material:**

The online version of this article (doi:10.1186/s12985-015-0459-1) contains supplementary material, which is available to authorized users.

## Background

Infectious salmon anaemia virus (ISAV) is an economically important pathogen of marine-farmed Atlantic salmon (*Salmo salar* L.). The disease infectious salmon anaemia (ISA) is arguably the most feared viral disease of the marine farmed salmon industry because it has continued to cause the Atlantic salmon farming industry severe economic losses in an increasing number of countries for the past 30 years. ISAV is the only species of the genus *Isavirus*, and one of the seven genera of the family *Orthomyxoviridae* that includes the influenza viruses [1–3]. A complete sequence of PB1 gene of a putative koi carp orthomyxovirus was obtained from koi carp in California with 43 % amino acid sequence identity with ISAV [4], and there is also an independent reference to an orthomyxovirus from koi carp [5], and to unknown viruses with morphology consistent with members of family *Orthomyxoviridae* isolated from baitfish in Wisconsin, USA [6]. The taxonomic status of these findings is not known. ISAV occurs in two basic genotypes, North American and European [7, 8]. The European genotype is more widespread [9] and shows greater genetic variation [10, 11] and greater virulence variation [12–14] than the North American genotype. ISA outbreaks have only been reported in farmed Atlantic salmon, although ISAV has been detected by RT-PCR in wild fish (Table 1). It is recognized that asymptomatically ISAV-infected fish exist [15, 16]. Since 2012, only Norway, Canada and Chile have reported ISA outbreaks. The ISA outbreaks reported in Canada have occurred in the Atlantic Ocean in New Brunswick, Nova Scotia, and Newfoundland and Labrador [17].

**Table 1:** Timeline (chronological history) of the detection of ISAV in wild fish related to first-time outbreaks of ISA in farmed Atlantic salmon

Year of sample & Test used	Country (location)	Wild fish species with ISAV (reference)	First-time outbreaks of ISA in farmed Atlantic salmon in country (reference)
1998-1999, Virus Isolation & RT-PCR	UK (Scotland)	Sea trout, Brown trout, Atlantic salmon [61]	Scotland, UK in 1998 [47]
2000, RT-PCR	Canada (New Brunswick)	Salmonids [62]	New Brunswick, Canada in 1996 [63]
2000, RT-PCR	UK (Scotland)	Atlantic salmon [49]	
2000, RT-PCR	UK (Scotland)	Sea trout, Brown trout, Atlantic salmon [64]	
2001, RT-PCR	West Greenland fishery	Atlantic salmon [65]	
2001, RT-PCR	USA (Maine)	Atlantic salmon (P. Barbash, cited by [66])	Maine, USA in 2001 [67]
2000-2002, Virus Isolation & RT-PCR	USA (Maine)	Pollock*, Atlantic cod** [66]	
1998; 2001–2003, RT-PCR	Norway (western Norway)	Salmonids (wild trout, Atlantic salmon) [31]	Norway in 1984 [68]
1995-2002, Antibody ELISA	USA (Maine & Massachusetts)	Atlantic salmon [69]	
2010, RT-PCR	Denmark	Atlantic salmon^§^ [70]	
2010, RT-PCR	Chile (an estuary in southern Chile)	free-living *Salmo salar* (escapees) [15]	Chile in 2007 [71]
	Faroe Islands, Denmark		Faroe Islands, Denmark in 2000 [72]

*Pollock taken from inside a marine cage with ISA-disease salmon was weak RT-PCR positive;

**Atlantic cod taken from a well boat holding salmon from a marine cage with clinically diseased fish was CPE positive on SHK cell culture.

^§^Danish salmon produced for restocking purposes.

ISAV has a segmented genome with eight single-stranded RNA segments of negative polarity [1]. The *Orthomyxoviridae* family is known to exhibit high mutation rates, and ISAV occurs in at least 30 recognized HPR variants [9, 18]. When viruses mutate, ‘drift variants’ arise that can escape detection by real-time RT-qPCR tests due to mismatches in the primer-probe binding sites [19]. When a mutation occurs in the precise region that a given primer or probe was designed to anneal, test reliability can be significantly decreased [20] producing inconsistent positive and false-negative readings between replicates [21]. There is no scientific standard for interpretation of high, or inconsistent threshold cycle (*C*_t_) values, and so these kinds of results are interchangeably reported as “negative,” “suspicious” or “positive” [22, 23]. For the purposes of this work, we simply designated our results as negative or non-negative.

In Canada, a federally reportable fish disease such as ISA must be confirmed at the Fisheries and Oceans (DFO) Canada National Reference Laboratory [24] through successful virus isolation in cell culture [25]. However, ISAV-HPRΔ strains of low virulence and the non-pathogenic ISAV-HPR0 strains grow poorly or not at all in currently available fish cell lines [15, 26–29]. Gagné and Ritchie [30] report an increasing number of ISAV positive results by RT-PCR in Canada that cannot be confirmed by other diagnostic tests. It is also recognized in Norway that ISAV may be present even when attempts at virus isolation are negative as ISAV has never been isolated from a wild salmon despite positive RT-PCR results (Table 1) [31].

While virus isolation is considered the “gold standard” for virus identification [32], it can produce “false negative results” [20]. Virus isolation requires tissue heavily infected with intact virus [33], which is unlikely to be found in wild salmon which are culled by predators that target weakened fish [34]. As well, intact, infective ISAV may not reliably occur in healthy salmon that have been harvested for several days, such as fish found in markets. Molecular tests, however, have the capacity to detect low levels of virus fragments [35] making them ideally suited for the types of samples available to this study.

There is no universally accepted ISAV RT-qPCR TaqMan® assay. Most diagnostic laboratories use the Snow et al. [36] primer-probe set targeting a 104 bp-fragment on ISAV segment 8 [37, 38]. Some laboratories and researchers have found the Plarre et al. [31] primer-probe set targeting ISAV segment 7 to be more sensitive. Other researchers have published different ISAV segment 8 primer-probe sets that are highly sensitive [13], but are not included in the OIE Manual [38]. There is also a long standing conventional RT-PCR protocol targeting ISAV segment 8 using a primer set initially developed by Devold et al. [39], which is less sensitive than real time RT-qPCR. This yields a PCR product of 221 bp, which includes the first 94 bp of the 104 bp-PCR amplicon of the Snow et al. [36] RT-qPCR TaqMan® assay, with the reverse primer sequences of both assays overlapping in 9 nucleotides. While preliminary results from this study were interpreted as controversial [40, 41], they are consistent with the nature of both the tests and the samples, i.e., wild fish and fish from markets. The findings in the present study are supported by the many unpublished ISAV RT-PCR positive results in farmed and wild salmon in British Columbia, which exist as unpublished federal laboratory exhibits released by the Cohen Commission into the Decline of the Sockeye Salmon of the Fraser River [42]. Here we present more complete test results demonstrating that the ISAV sequences detected in British Columbia (BC) fish, both ISAV-HPRΔ and ISAV-HPR0, are of European genotype, with a mismatch in segment 8 that contributes to the inconsistent results of the RT-qPCR TaqMan® assay, and represents a new ISAV variant that appears to occur in BC in absence of high losses to the salmon farming industry. It would add to the knowledge of ISAV to test fresh moribund farmed salmon using the methods we describe here.

## Results and Discussion

### Sample RNA quality was based on real-time RT-PCR for ELF-1α as internal control for all samples

**Fig. 1:**
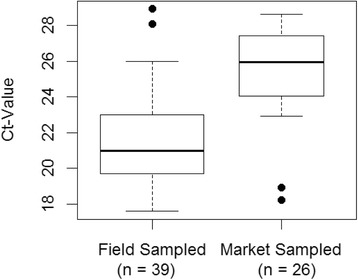
Boxplots highlighting the systematic difference in the *Ct*-values for the market-sampled vs. field-sampled fish. The bold, middle line represents the median for each group; the box covers the range between the lower and upper quartiles; the whiskers extend to the last value still within 1.5 times the interquartile arrange of the relevant quartile; individual points beyond these are plotted individually as potential outliers.

The ELF-1α controls showed a considerable variation between samples (within and between species). Fig. 1 highlights the systematic difference in the threshold cycle (*C*_t_) values for the market-sampled vs. field-sampled fish. It also highlights two outliers in each of the two groups. The log sheets for the two anomalously large values in the field-sampled group indicate potential delays in sample processing under suboptimal conditions. The anomalously low values in the market-sampled group came from a single shopping event. This in turn suggests that these fish were perhaps atypically fresh. Both these sets of outliers point to the potential for differences between sampling events to account for a substantial portion of the variability in these *C*_t_ values. A formal analysis of a mixed-effects model for these values using the R package, ‘lme4’ [43], provided the following estimates: (i) that the mean *C*_t_ values for the market-sampled group was 2.94 units higher than the mean for the field-sampled group, and (ii) that the standard deviation of the within-sampling-event means was 2.57, and that the residual deviation was only 1.47. Although the data set was too small to provide precise estimates of these parameters, it appears that most of the variability in the *C*_t_ values can be accounted for by variation in the sample quality – much of it unavoidably associated with the necessity to rely on (i) market purchases of farmed fish and (ii) logistics of sampling remote regions of British Columbia.

### Fewer than 2.0 % of British Columbia fish tested were “non-negative” in the real time RT-qPCR TaqMan® assay for ISAV

In the present study, we used the Snow et al. [36] primer-probe set targeting segment 8 with a cut-off *C*_t_ value established as the mean *C*_t_ value in the highest virus dilution for which all 30 replicates were positive (Additional file 1: Table S1). Thus for the purposes of this study, samples were considered “non-negative” when the fluorescence signal increased above the *C*_t_, and if the *C*_t_ value was ≤ 34.20. Samples with *C*_t_ > 34.2 to ≤ 39.9 were considered weak “non-negative” and > 40, suspicious as *C*_t_ of the last five cycles has higher uncertainity. Where the *C*_t_ value was zero, the result was deemed to be negative.

A total of 1,106 tissue samples were collected from market-sourced farmed salmon (397) and wild fish (708) obtained from (i) saltwater commercial fisheries, (ii) freshwater and saltwater sport fisheries, (iii) research sampling of juvenile salmon in seawater, and (iv) spawning adult salmon in freshwater habitat throughout British Columbia, Canada. As well, a sea louse (*Lepeophtheirus salmonis*) removed from a juvenile sockeye salmon was sampled. All samples were tested for ISAV using published real time RT-qPCR and/or conventional RT-PCR. The test results are detailed in Additional file 2: Table S2 and summarized in Table 2. Out of the total of 1,106 tissue samples, 20 (1.81 %) tested had a *C*_t_ value (Table **2**). Of these, only one sample (Fish # VR5, a spawning chum salmon in freshwater) was also positive in conventional RT-PCR with segment 8 and segment 6 HPR primers. One sample (Fish # TT48, an Atlantic salmon from a market) was also positive in conventional RT-PCR with segment 8, and two samples (Fish# SK20 and TT51, both Atlantic salmon from markets) were also positive in conventional RT-PCR with segment 6 HPR primers. The percentage of samples with a *C*_t_ value was more than two-fold greater in farmed fish tissues (2.77 %) compared to wild fish tissues (1.3 %). There were 56/65 fish that produced positive conventional RT-PCR results with no RT-PCR *C*_t_ values. All virus isolation attempts on these samples using ASK-2 and SHK-1 cell lines were negative. From a Canadian regulatory perspective, diagnostic confirmation requires virus isolation on permissive fish cell lines and virus identification [25] - hence a sample with a *C*_t_ value or positive conventional RT-PCR in this study was designated as “non-negative”.

**Table 2:** Number of samples for each species that (i) tested non-negative for infectious salmon anaemia virus (ISAV) by RT-qPCR^1^ and that produced sequences by conventional PCR for (ii) segment 6 and (iii) segment 8

Fish species		Farmed fish				Wild fish			
Common name	Scientific name	n	RT-qPCR	Conventional PCR seg. 8 sequence	Conventional PCR seg. 6 sequence	n	RT-qPCR	Conventional PCR seg. 8 sequence	Conventional PCR seg. 6 sequence
Atlantic salmon	*Salmo salar*	334	9	18	13^2^				
Chinook salmon	*Oncorhynchus tshawytscha*	13	0	0	0	102	2	2	0
Coho salmon	*Oncorhynchus kisutch*	4	0	0	0	68	1	6	1
Sockeye salmon	*Oncorhynchus nerka*					256	3	7	2
Kokanee	*Oncorhynchus nerka*					1	1	1	Na
Pink salmon	*Oncorhynchus gorbuscha*					118	0	2	0
Chum salmon	*Oncorhynchus keta*					68	1	1	1
Steelhead trout	*Oncorhynchus mykiss*	46	2	3	0	21	0	Na	Na
Cutthroat trout	*Oncorhynchus clarkii*					18	0	8^3^	13
Chum mackerel	*Scomber japonicus*					13	0	1	0
Pacific herring	*Clupea pallasi*					44	0	1	0
Sea louse	*Lepeophtheirus salmonis*					1	1	0	0
Total		397	11	21	13	709	9	29	17

^1^All fish tissue samples were screened by the real time RT-qPCR TaqMan® assay for ISAV. However, only some of the samples were also tested in conventional RT-PCR for segment 6 HPR or segment 8. Therefore, the numbers do not reflect a direct comparison of the 3 different RT-PCR assays. Na denotes Not applicable.

^2^2 others with a PCR product but not sequenced.

^3^5 others with a PCR product but not sequenced.

### ISAV sequences detected in British Columbia fish have a mismatch in segment 8 compared to classical ISAV and represent a new ISAV variant of European genotype

Whereas all fish tissue samples were screened by the real time RT-qPCR TaqMan® assay for ISAV, only a portion of these samples was additionally tested by conventional RT-PCR for segment 8 or segment 6 HPR. Table 2 lists all non-negative test results by species and by farmed vs. wild status. This study did not attempt a direct comparison of the 3 different RT-PCR assays. Such an effort would require standardizing sample quality, which would require direct access to salmon in the farms.

**Fig. 2:**
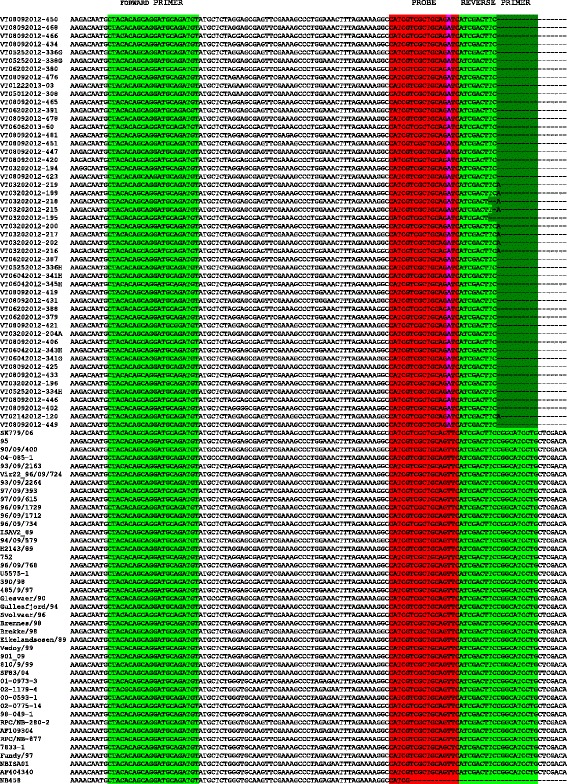
Alignment of ISAV segment 8 nucleotide sequences in the target region of the primer-probe set of the real time RT-qPCR TaqMan® assay for ISAV [36]. The ISAV sequences belonged to a 221 bp-long PCR product amplified from 50 samples in this study and 47 selected classical ISAV segment 8 sequences of different ISAV isolates in the GenBank database. The nucleotide sequences were aligned using CLUSTAL X with the default settings [58]. Green is forward primer and reverse primer sequences and red is probe sequence. The single nucleotide mutation in the probe sequence is in blue. The reverse primer sequence includes the 9-nucleotide overlap between the reverse primer sequence of the real time RT-qPCR TaqMan® assay [36] and that of the conventional RT-PCR protocol [39]

To determine the genetic relationship between the ISAV sequences in this study and ISAV strains worldwide, we compared the segment 8 sequences using multiple alignment and phylogenetic analysis. All the 50 sequences from this study aligned well in a 221 bp-long fragment with 47 selected classical ISAV segment 8 sequences of different ISAV isolates in GenBank (GenBank Database) (Fig. 2). This alignment revealed a consistent single nucleotide mutation (5’-CAT CGT CGC TGC AGA TC-3’) in the 3’ region of the probe sequence (5’-CAT CGT CGC TGC AGT TC-3’) [36]. This mutation in the BC samples would contribute to the apparent failure of the real time RT-qPCR TaqMan® assay for ISAV in the 49 samples, positive in segment 8, but with no *C*_t_ value in the real time RT-qPCR TaqMan® ISAV assay. None of the classical ISAV segment 8 sequences in the GenBank database have this mutation. While a single nucleotide variation is a minor mutation, its placement in a region that an ISAV probe sequence seeks to anneal in a standard OIE ISAV test, makes this a significant mutation that warrants recognition as a new variant. Improvements aimed at better detection of this variant are currently being developed. Recognition of, and testing for, this variant are essential prerequisites for determining how widespread it is.

**Fig. 3:**
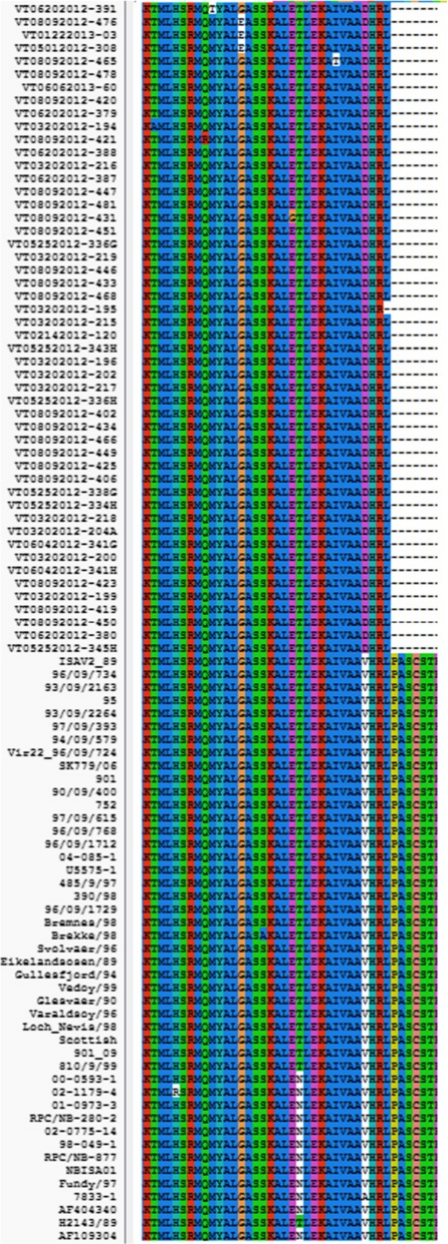
Alignment of ISAV segment 8 amino acid sequences in the target region of the primer-probe set of the real time RT-qPCR TaqMan® assay for ISAV [36]. The ISAV sequences are those in Fig. 2. The amino acid sequences in the region between the two primers (Forward Primer and Reverse Primer) were aligned using CLUSTAL X with the default settings [58]. The single nucleotide mutation in the probe sequence resulted in a single amino acid change from V to D

The alignment of amino acid sequences in a 118 bp-long fragment of the same ISAV sequences in Fig. 2 without the primer sequences is shown in Fig. 3. The polypeptide aligned well. The number of mutations shown in Fig. 3 is slightly less than the number of mutations in Fig. 2. This is because of the redundancy nature of the genetic code. For example, the mutation from AAAGCCC to AAGGCCC in VT02142014-120 is not shown in Fig. 3; this is because both AAA and AAG are translated to K. The single nucleotide mutation in the probe sequence of Snow et al. [36] resulted in a single amino acid change from V to D and would therefore produce a functional full-length viral protein.

**Fig. 4:**
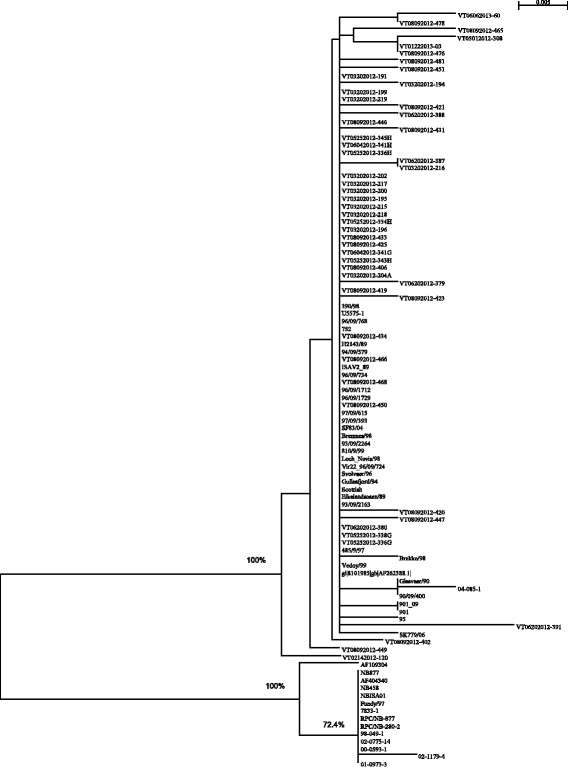
Phylogenetic tree showing the genetic relationship between the 97 ISAV sequences aligned in Fig. 2. Sequences were aligned and the phylogenetic tree was generated by using CLUSTAL X with the default settings [58]. Phylogenetic analysis using Neighbor-Joining bootstrap method (1000 replicates) provided satisfactory bootstrap support (bootstrapping values are shown for branch-points with greater than 70 % bootstrap support)

Phylogenetic analysis was used to further determine the genetic relationship between the newly discovered BC ISAV variant in the present study and the classical ISAV strains worldwide. Fig. 4 shows the phylogenetic tree generated with these sequences with satisfactory bootstrap support (bootstrapping values more than 70 % are marked). In addition to showing the relationship of the ISAV sequences from this study (all “VT” sequences), this tree also supports the well-established major division between North American genotype and European genotype ISAV. All the ISAV sequences detected in this study are of the European genotype. While these BC sequences tend to be similar to each other, their differences with other European sequences are very small. The apparent wide diversity of the BC sequences in the tree reflects the nature of the sampling, and the fact that there was no single-source-selected amplification as occurs with the classical ISAV isolates from disease outbreaks. Moreover, the branching in the tree also indicates that some (VT02142012-120, VT08092012-449, and VT08092012-402) diverted early from most of the classical European genotype sequences, while others (VT06062013-60, VT08092012-465, VT05012012-308, and VT06202012-391) have evolved separately. The data support the observation that ISAV can exist in a region for a period of years in absence of outbreaks and in a state that may not be detectable by methods designed to diagnose virulent outbreaks in moribund farmed fish.

### ISAV sequences detected in British Columbia fish include both ISAV-HPRΔ and ISAV-HPR0 and are of European genotype

The detection of ISAV-HPR0 in British Columbia fish (Fish# SK20) was designated a suspect result by the Canadian Food Inspection Agency (CFIA), because of the inability for follow up by the federal authorities. With the widespread occurrence of ISAV-HPR0 variants in many parts of the world and its potential as a precursor to the virulent strains of ISAV [13], it is essential that RT-PCR positive results based on segment 8 primers be followed up with conventional RT-PCR using segment 6 primers targeting the HPR. Sequencing of the PCR product is also essential in order to determine the ISAV HPR type present (ISAV-HPRΔ or ISAV-HPR0 or both) [9]. ISAV-HPR0 has only been reported in apparently healthy fish and has never been associated with clinical or pathological diagnosis of ISA disease [44].

Of the fish tested in conventional RT-PCR for segment 6 HPR, sequences of the PCR product were obtained from 13 farmed fish samples (13 Atlantic salmon) and 17 from wild fish samples (1 coho, 2 Sockeye, 1 Chum and 13 Cutthroat) (Table 2). In contrast to conventional RT-PCR for segment 8, where 49 samples positive in segment 8 had no *C*_t_ value in the real time RT-qPCR TaqMan® assay for ISAV, only 3 samples (Fish# SS132, MQ06, and P113, Additional file 2: Table S2) were positive in conventional RT-PCR for segment 6, with no *C*_t_ value. These samples were also negative by conventional RT-PCR for segment 8.

The sequences of ISAV segment 6 obtained from the PCR products matched ISAV-HPR5 on 29 samples, one had both ISAV-HPR5 and ISAV-HPR7b and one sample matched ISAV-HPR0 (Additional file 2: Table S2). All were of European genotype. ISAV-HPRΔ strains of HPR5 and HPR7b types have been associated with ISA outbreaks in Norway [45, 46], Scotland [47] and Chile [9, 48]. Thus our data indicate that ISAV-HPRΔ strains can be present without clinical disease in farmed fish and without being detected by virus isolation, which is in agreement with other reports [15, 16].

**Fig. 5:**
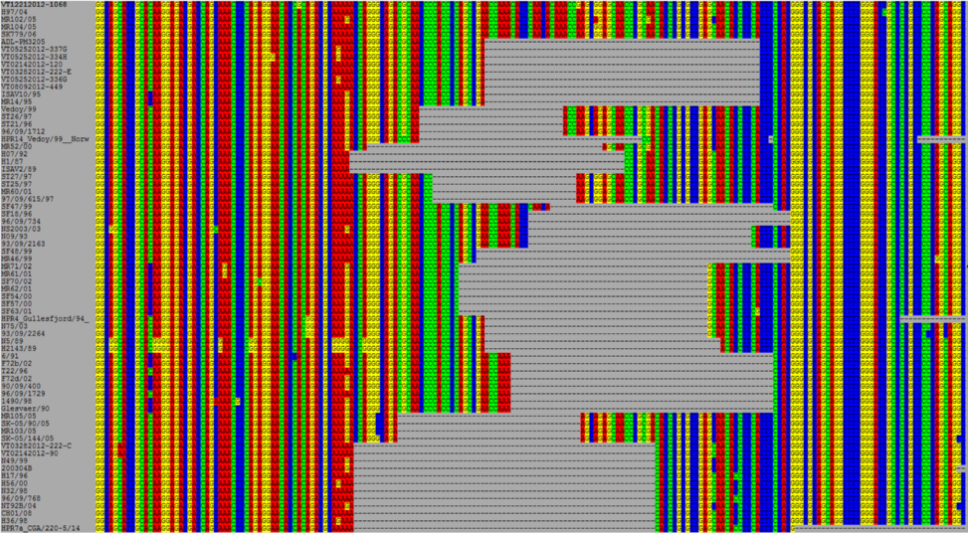
Alignment of ISAV segment 6 HPR nucleotide sequences containing the deletion. The alignment was adjusted manually as all known alignment software packages cannot align nucleotide sequences in such a delicate and complex area. Among 101 sequences analyzed, 39 of them are British Columbia isolates (ID starts with VT); the deletions of these 39 sequences are in three patterns. The first pattern is VT12212012-1068; the second pattern is VT03282012-222-C and VT02142012-90; the third pattern has 36 isolates and this pattern can be shown in VT05252012-337G. To save space in the figure, we used 6 sequences to represent the 36 sequences in this pattern

To determine the genetic relationship between the segment 6 HPR sequences detected in BC and worldwide, we compared the HPR sequences using multiple alignment and phylogenetic analysis. A total of 316 sequences aligned well and the phylogenetic tree was generated depicting the overall relationship among all ISAV isolates for which segment 6 sequence is available (data not shown). Virulent ISAV isolates have a deletion in segment 6 HPR sequence [44]. Of the 316 segment 6 HPR sequences, we only identified 101 sequences that were long enough to display this deletion. We aligned these 101 sequences to show the deletion. No existing alignment software packages can align nucleotide sequences in this delicate and complex area, thus the alignment has been manually adjusted. When we prepared a figure to show these sequences and the deletion, we found it is hard to show so many sequences in a figure so that some of the sequences that behave the same in this deletion area were removed. Fig. 5 shows a portion of the alignment containing the deletion. In this figure, 71 sequences were included, representing the 101 sequences. The first five sequences of the alignment, including VT12212012-1068, are complete, i.e., they have no deletion and belong to ISAV-HPR0 [49]. The rest of the sequences have a deletion and belong to ISAV-HPRΔ [11, 44].

**Fig. 6:**
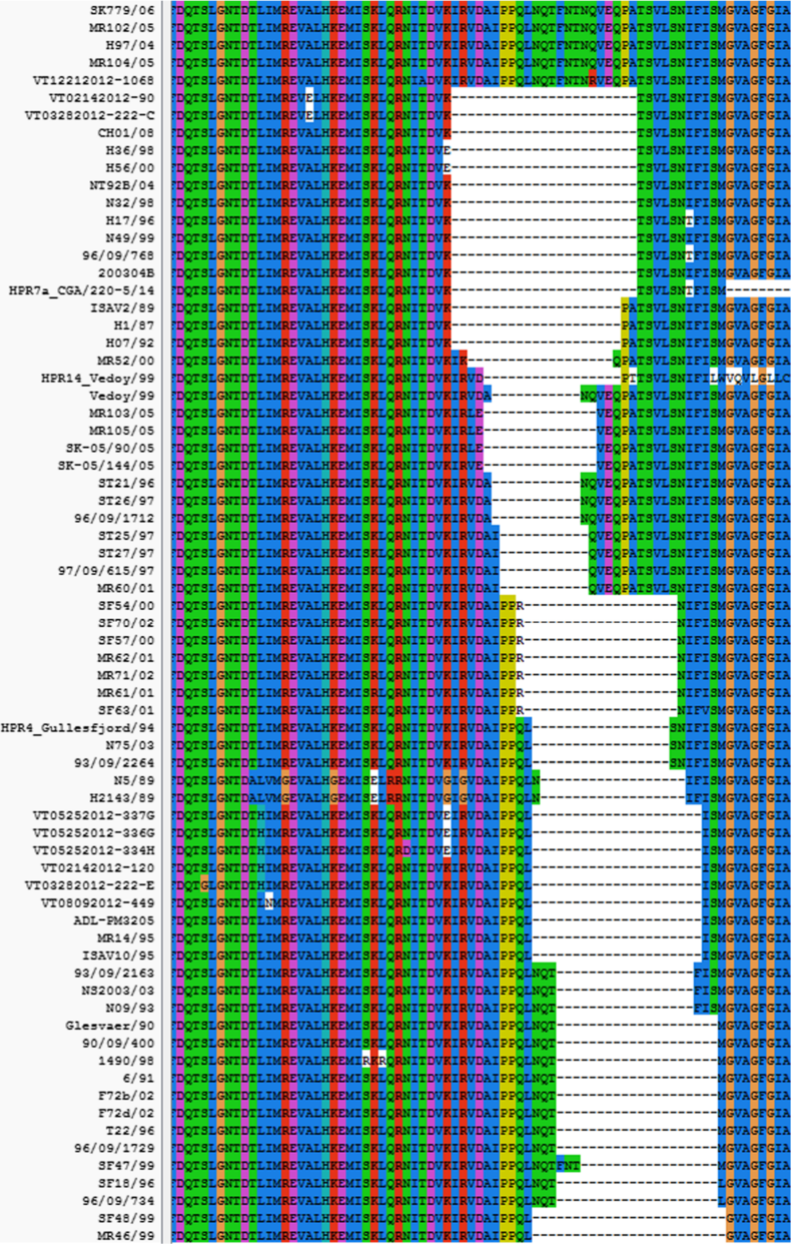
Alignment of ISAV segment 6 HPR amino acid sequences containing the deletion. The alignment was adjusted manually as all known alignment software packages cannot align amino acid sequences in such a delicate and complex area in Fig. 5. The alignment confirms the three ISAV HPR patterns in Fig. 5: The first pattern VT12212012-1068 belongs to ISAV-HPR0; the second pattern VT03282012-222-C and VT02142012-90 belongs to ISAV-HPR7b; the third pattern with 36 isolates and shown in VT05252012-337G belongs to ISAV-HPR5. Similarly to Fig. 5, only 6 of the 36 sequences in the third pattern are included in Fig. 6

The alignment of amino acid sequences of the ISAV segment 6 HPR sequences in Fig. 5 without the primer sequences is shown in Fig. 6. Both figures show extensive deletions in the HPR. Existing software cannot produce high-quality alignments in areas with deletions. Thus this alignment was manually adjusted to reveal the deletion event. The deletions in HPR probably occur through homologous recombination (copy-choice recombination, presumably because of strand-switching by the viral RNA polymerase [50] during negative RNA strand synthesis from one nucleic acid template of one virus to another. Fig. 6 confirms the three ISAV HPR types found in the BC samples: ISAV-HPR0 (VT12212012-1068), ISAV-HPR7b (VT03282012-222-C and VT02142012-90), and ISAV-HPR5 (VT05252012-337G and 5 other samples) and also reveals the relationship between these HPR types and ISAV HPR types in other countries.

**Fig. 7:**
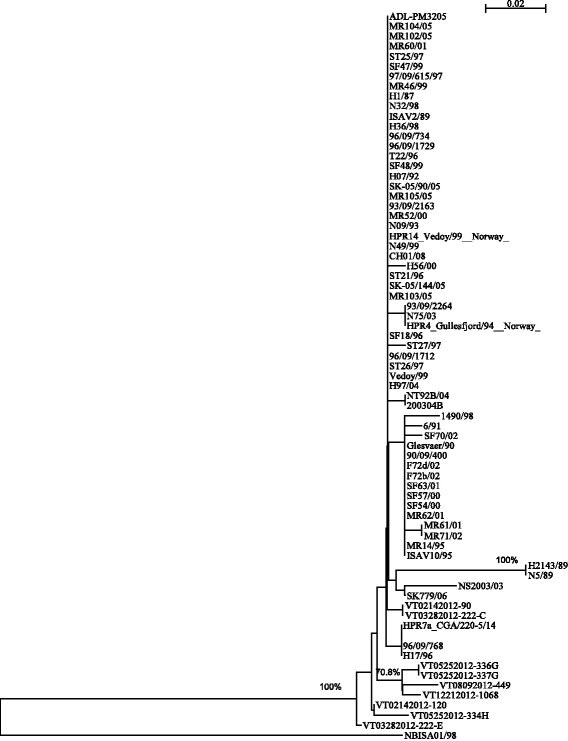
Phylogenetic tree showing the genetic relationship between the 71 representative segment 6 HPR sequences aligned in Fig. 4. Sequences were aligned and the phylogenetic tree was generated by using CLUSTAL X with the default settings [58]. Phylogenetic analysis using Neighbor-Joining bootstrap method (1000 replicates) provided satisfactory bootstrap support (bootstrapping values are shown for branch-points with greater than 70 % bootstrap support)

Fig. 7 shows the phylogenetic tree generated with the sequences in Fig. 4 with satisfactory bootstrap support (bootstrapping values more than 70 % are marked); where NBISA01/98 is used as an outgroup. This tree contains only the isolates from Fig. 5. It was not created to reflect the whole evolutionary history of ISAV segment 6, but to help with the analysis of the HPR deletion. Comparing Figs. 5, 6 and 7, we can find some consistency, i.e. isolates that show the same deletion pattern tend to be closer inside the tree. Although such consistency only exists for a few groups, the combination of these two approaches may reveal more insights on ISAV’s evolutionary history.

## Conclusions

To our knowledge the present work constitutes the first published report of the detection of ISAV sequences in fish from British Columbia, Canada. The sequences detected, both of ISAV-HPRΔ and ISAV-HPR0 are of European genotype. The virus in these samples has a mismatch in segment 8 that can account for failure of the real time RT-qPCR TaqMan® assay for ISAV recommended in the OIE Aquatic Manual. Furthermore, these sequences are different from the classical ISAV segment 8 sequences, and this difference suggests the presence of a new ISAV variant of European genotype in BC. Our results further suggest that ISAV-HPRΔ strains can be present without clinical disease in farmed fish and without being detected by virus isolation using fish cell lines. Recent reports on ISAV surveillance in Washington, USA [41], and in British Columbia [17] report no ISAV detection. However, neither of these studies report on samples from the known target host of ISAV, farmed Atlantic salmon, and it is unreported whether weak RT-PCR positives similar to ours were found, and interpreted as “negative”. More research on the source of this variant ISAV sequence is critically important for assessing the risks to both farmed and wild salmon in the region, its origin and to better understand ISAV evolution.

## Methods

### Sampling

Wild fish were collected from freshwater spawning grounds, fresh and saltwater sport fisheries, saltwater commercial fisheries, and saltwater scientific fisheries. Wild fish samples included all species of Pacific salmon (*Oncorhynchus* sp.), Atlantic salmon (*Salmo salar*), steelhead (*Oncorhynchus mykiss*), cutthroat trout (*Oncorhynchus clarkii*), kokanee (*Oncorhynchus nerka*), Pacific chub mackerel (*Scomber japonicus*) and Pacific herring (*Clupea pallasi*) (Table 2). All fish and the sampled organs were photographed *in situ*. Gill and heart were sampled from the whole wild fish. Gill and remnant head kidney were sampled from the gutted, head-on farmed salmon and also the farmed salmon heads purchased from markets. The hearts were not available from these samples. All samples were placed immediately in sterile Whirl-Pak® bags (Nasco Inc., Fort Atkinson, WI) on ice with replicate samples preserved in RNALater® (Ambion Inc., Foster City, CA) and shipped overnight by courier to the testing laboratory. At the laboratory, samples were immediately stored at −80 °C until they were analyzed. The testing laboratory ran tests exclusively on the samples and did not participate in the collection of the samples or in the custody of the samples prior to receipt of the samples.

### Total RNA preparation

Total RNA was isolated using a modified total RNA extraction protocol with the RNeasy® mini Kit (QIAGEN). Briefly, each tissue (or pool of tissues) was weighed and macerated to a 10 % suspension w/v in phosphate buffered saline (PBS) with 10x antibiotics. The specimen supernatant was used for RNA extraction. Samples preserved in RNAlater® were first washed three times with PBS and then homogenized as described above prior to total RNA extraction. Total RNA was isolated from samples using 1.25 ml of TRIZOL Reagent (Invitrogen) and 375 μl of sample volume as previously described [51]. The Viral RNA mini Kit (QIAGEN) was also utilized on selected samples following the manufacturer’s recommended protocol. In all cases, the extracted RNA was eluted in 20–50 μl of nuclease-free water, and RNA yields were quantified and purity analysed using the OD260/280 ratio and a NanoDrop ND-1000 spectrophotometer (Thermo Fisher Scientific). The eluted RNA was tested immediately following quantitation, or was stored frozen at −80 °C prior to use in RT-PCR.

### Real-time RT-qPCR

RT-qPCR was run on the LightCycler 480 (Roche Applied Science), version 4.0. The crossing point (Cp) or threshold cycle (*C*_t_) was determined by use of the maximum-second-derivative function on the LightCycler software release 1.5.0. The Roche LightCycler® 480 RNA master Hydrolysis Probe kit (Roche Diagnostics) was employed for all RT-qPCR reactions according to the manufacturer’s specifications. Sample RNA quality was based on RT-qPCR for elongation factor 1 alpha (ELF-1α) as internal control targeting either Atlantic salmon ELF-1α (GenBank accession number BT072490) or Chinook salmon ELF-1α (GenBank accession number FJ890356) using primers, probes, and RT-qPCR thermal cycling parameters as previously reported [52]. RNA quality varied, with the higher *C*_t_ values generally occurring in farmed salmon from markets where the interval between harvest and sampling was on the order of days, not minutes as was the case for most wild fish samples. Nonetheless, some wild fish, such as the cutthroat trout and LaP1, were caught by fishermen with unavoidable delays in processing the sampled fish. Such delays may have contributed to the higher *C*_t_ values in some of these samples. Results from tests with *C*_t_ values above 40 or at 0 were designated as negative. In addition, these samples would be considered unfit for further testing if after re-extraction and repeated RT-qPCR the same results were obtained.

Detection of ISAV with the one-step real-time RT-qPCR [51] was carried out using the primer-probe set developed by Snow et al. [36] targeting segment 8 and described in the OIE Aquatic Manual [38]. However, there is no defined *C*_t_ value cut off to aid interpretation of results. In this study, the cut-off *C*_t_ value for this probe was set at ≤34.20 ± 1.05 based on 10-fold dilutions of cell culture ISAV (ADL 2007) each tested in 5 replicates and repeated 6 times for a total of 30 replicates, and denotes the mean *C*_t_ value in the highest virus dilution for which all 30 replicates were positive (Additional file 1: Table S1). The same preparations were also tested in the conventional RT-PCR methods below, allowing for a correlation of the cut-off *C*_t_ value with the conventional RT-PCR tests on cell culture virus.

### Conventional RT-PCR and nucleic acid sequencing

Samples testing positive by real-time RT-qPCR were further tested using conventional one step RT-PCR targeting segments 6 and 8 to obtain PCR products for DNA sequencing. The ISAV conventional one step RT-PCR used ISAV-specific primers FA-3/RA-3 and the conditions described by Devold et al. [39] for RNA segment 8, and the following primers segment 6 HPR primers, Fwd 5'-GCC CAG ACA TTG ACT GGA GTA G-3', and Rev 5'-AGA CAG GTT CGA TGG TGG AA-3' described by Kibenge et al. [9] for RNA segment 6, and was run in a Bio-Rad thermal cycler (Bio-Rad). Briefly, amplification was performed using 50 μl reaction mixture utilizing One-step RT-PCR kit (QIAGEN) as follows: the reaction mixture contained 2 μl of total RNA, 10 μl of 5X QIAGEN OneStep RT-PCR buffer, 2 μl of dNTPs, 10 units of RNAse inhibitor (Life Technologies), 0.6 μM (final concentration) of each primer pair, and 2 μl of QIAGEN OneStep RT-PCR enzyme mix in a final volume of 50 μl. The RT-PCR amplification conditions were 1 cycle at 50 °C for 30 min, one cycle at 95 °C for 15 min, 40 cycles at 94 °C for 30 s, 60 °C for 60 s and 72 °C for 90 s and 1 cycle at 72 °C for 10 min before soaking at 4 °C. Amplified products were analyzed by electrophoresis on 1 % agarose gel and purified using High Pure PCR Product Purification Kit (Roche). The PCR products were cloned into the pCRII vector using a TOPO TA cloning kit (Invitrogen) in preparation for nucleotide sequencing, although in some cases the RT-PCR products were sequenced directly without cloning. Plasmid DNA for sequencing was prepared as per Kibenge et al. [53], and DNA sequencing as per Kibenge et al. [10] by ACGT Corporation (Toronto, Ontario, Canada). Sequence analysis used the BLAST programs [54] against the latest release at GenBank [55], the Sequence Manipulation suite version 2 [56], and the FASTA program package for microcomputers [57]. Sequences are available through GenBank and their accession numbers are listed in Table 3.

**Table 3:** GenBank Accession numbers used in the multiple alignments and phylogenetic analyses and of new sequences from this study

Isolate or sample ID	Segment 6	Segment 8	Reference
VT02142012-120	JQ857081	JQ857078	This study
VT05252012-334H	KR998473	KR998431	This study
VT05252012-336G	KR998474	KR998432	This study
VT08092012-449	KR998475	KR998433	This study
ISAV2/89	DQ785246		
96/09/734	DQ785250	DQ785278	
96/09/768	DQ785249	DQ785277	
96/09/1729	DQ785251	DQ785279	
96/09/1712	DQ785245	DQ785273	
93/09/2264	DQ785255	DQ785283	
93/09/2163	AF427049	DQ785281	
HPR4_Gullesfjord/94_Norway	AF302801	AF262384	
HPR14_Vedoy/99_Norway	AF302803	AF262383	
Glesvaer/90	AF220607	AF262382	
90/09/400	DQ785248	DQ785276	
H2143/89	DQ785247	DQ785275	
SK779/06	EU118820	EU118822	
NBISA01/98	AF283996	AF315063	
VT03282012-222-C	KR998476		This study
VT03282012-222-E	KR998477		This study
VT05252012-337G	KR998478		This study
VT12212012-1068	KR998479		This study
ADL-PM3205	HQ011267		
MR104/05	DQ108607		
MR102/05	DQ108605		
MR60/01	AY127876		
ST25/97	AF364885		
SF47/99	AF364888		
97/09/615/97	DQ785252		
MR46/99	AF364896		
HI/87	AF364893		
N32/98	AF364883		
H36/98	AF302799		
T22/96	AF364889		
SF48/99	AF364878		
H07/92	AF364898		
SK-05/90/05	FM203287		
MR105/05	DQ108608		
MR52/00	AF364892		
N09/93	AF364895		
N49/99	AF364876		
CH01/08	EU851043		
H56/00	AF364880		
ST21/96	AF364886		
SK-05/144/05	FM203274		
MR103/05	DQ108606		
N75/03	AY971661		
SF18/96	AF364869		
ST27/97	AF364897		
ST26/97	AF364879		
H97/04	DQ108604		
NT92B/04	AY973188		
200304B	FM203244		
1490/98	AF391126		
6/91	AF364894		
SF70/02	AY127880		
F72d/02	AY971657		
F72b/02	AY971656		
SF63/01	AY127879		
SF57/00	AF364890		
SF54/00	AF364884		
MR62/01	AY127878		
MR61/01	AY127877		
MR71/02	AY127881		
MR14/95	AF364873		
ISAV10/95	DQ785254		
N5/89	AY127882		
NS2003/03	AY973182		
HPR7a_CGA/220-5/14	KJ944288		
96/09/768	DQ785249		
H17/96	AF364891		
VT03202012-194		KR998434	This study
VT03202012-195		KR998435	This study
VT03202012-196		KR998436	This study
VT03202012-199		KR998437	This study
VT03202012-200		KR998438	This study
VT03202012-202		KR998439	This study
VT03202012-204A		KR998440	This study
VT03202012-215		KR998441	This study
VT03202012-216		KR998442	This study
VT03202012-217		KR998443	This study
VT03202012-218		KR998444	This study
VT03202012-219		KR998445	This study
VT05012012-308		KR998424	This study
VT05252012-336H		KR998425	This study
VT05252012-338G		KR998446	This study
VT06042012-341G		KR998447	This study
VT06042012-341H		KR998448	This study
VT06042012-343H		KR998449	This study
VT06042012-345H		KR998450	This study
VT08092012-402		KR998451	This study
VT08092012-406		KR998452	This study
VT08092012-419		KR998453	This study
VT08092012-420		KR998454	This study
VT08092012-421		KR998455	This study
VT08092012-423		KR998456	This study
VT08092012-425		KR998457	This study
VT08092012-431		KR998426	This study
VT08092012-433		KR998427	This study
VT08092012-434		KR998458	This study
VT08092012-446		KR998428	This study
VT08092012-447		KR998459	This study
VT08092012-450		KR998460	This study
VT08092012-451		KR998461	This study
VT08092012-465		KR998462	This study
VT08092012-466		KR998463	This study
VT08092012-468		KR998464	This study
VT08092012-476		KR998465	This study
VT08092012-478		KR998466	This study
VT08092012-481		KR998467	This study
VT06202012-379		KR998468	This study
VT06202012-380		KR998469	This study
VT06202012-387		KR998470	This study
VT06202012-388		KR998429	This study
VT06202012-391		KR998471	This study
VT01222013-03		KR998430	This study
VT06062013-60		KR998472	This study
Vir22_96/09/724		DQ785286	
97/09/393		DQ785284	
97/09/615		DQ785280	
94/09/579		DQ785285	
SF83/04		AY744395	
Svolvaer/96		AF262381	
Bremnes/98		AF262385	
Brekke/98		AF262380	
Eikelandsosen/89		AF262386	
810/9/99		DQ022085	
95		DQ785282	
901		GU830910	
752		GU830902	
390/98		DQ003602	
485/9/97		DQ003605	
U5575-1		DQ003603	
04-085-1		DQ058660	
01-0973-3		DQ003607	
02-1179-4		DQ003601	
00-0593-1		DQ003606	
02-0775-14		DQ003604	
98-049-1		DQ003600	
RPC/NB-280-2		AF312317	
AF109304		AF109304	
RPC/NB-877		AF312316	
7833-1		AF312315	
Fundy/97		AF262389	
AF404340		AF404340	
NB458		AY151798	

### Phylogenetic analyses

Sequences were aligned and phylogenetic trees were generated using CLUSTAL X with the default settings [58]. Alignment regions containing gaps were excluded from the analysis. The results were analyzed by using the bootstrap method (1000 replicates) to provide confidence levels for the tree topology. We then used different outgroup sequences to determine and verify the root of each tree.

### Virus isolation

Primary virus isolation was attempted on some of the RT-PCR “non-negative” samples using Salmon head kidney (SHK-1 and ASK-2) cell line monolayers. SHK-1 [59] and ASK-2 cells [39] were grown as previously described [12]. Homogenized tissues were inoculated on monolayers of SHK-1 and/or ASK-2 cell lines following standard protocols in the OIE Aquatic Manual [38]. Briefly, each tissue was weighed and macerated to a 10 % homogenate w/v in PBS with 10x antibiotics. The homogenates were centrifuged at 205.3 g for 15 min at 4 °C. The supernatants were individually filtered using 0.45 μM syringe filters to remove any bacteria prior to use in virus isolation attempts. 24 hr-old cell monolayers in tissue culture flasks free of medium were inoculated with the sample supernatant diluted 1:10 in serum-free medium, and incubated for 2 hr at room temperature to allow for virus adsorption. Maintenance medium was then added and the inoculated cells were then incubated at 16 °C and infection was allowed to proceed with daily monitoring using an inverted light microscope until the CPE was evident or 21 days and the flasks were frozen at −80 °C. Virus isolation was monitored by RT-PCR on the cell lysates since virus replication may occur without development of apparent CPE [60]. CPE negative and RT-PCR negative cultures were passaged on fresh cell monolayers. A sample was considered negative if no CPE or positive RT-PCR was observed after three blind passages.

### Ethics

The *in vitro* work was approved by the UPEI Biosafety Committee.

## Additional files

Additional file 1:
**Correlation of mean Ct value with conventional RT-PCR with ISAV segment 8 and HPR primers on cell culture virus.** Table showing Correlation of mean *C*_t_ value with conventional RT-PCR with ISAV segment 8 and HPR primers on cell culture virus. (DOC 33 kb) Additional file 2:
**Fish tissue samples testing “non-negative” for infectious salmon anaemia virus (ISAV) from** **2012-2013** ^**1**^ **.** Table listing the ISAV segment 8 tests done in replicates, which were in some cases repeated. Values in this column represent how many replicates produced a *C*_t_ value, or the averaged result of tests. (DOC 117 kb)
