# Supplementary material for: Discovery of variant infectious salmon anaemia virus (ISAV) of European genotype in British Columbia, Canada
Source: Virol J. 2016 Jan 6;13:3. doi: 10.1186/s12985-015-0459-1 (PMC4702313; doi:10.1186/s12985-015-0459-1)
Supplement: Additional file 1: — Correlation of mean Ct value with conventional RT-PCR with ISAV segment 8 and HPR primers on cell culture virus. Table showing Correlation of mean C t value with conventional RT-PCR with ISAV segment 8 and HPR primers on cell culture virus. (DOC 33 kb) [file 12985_2015_459_MOESM1_ESM.doc]

**Supplementary Table S1. Correlation of mean *C*_t_ value with conventional RT-PCR with ISAV segment 8 and HPR primers on cell culture virus.**

|  | **ISAV dilution** | | | | | | | | |
| --- | --- | --- | --- | --- | --- | --- | --- | --- | --- |
|  | Neat | 10^-1^ | 10^-2^ | 10^-3^ | 10^-4^ | 10^-5^ | 10^-6^ | 10^-7^ | 10^-8^ |
| Snow probe | 17.95±0.44  (30/30) | 20.69±0.25  (30/30) | 24.40±0.47  (30/30) | 28.07±0.84  (30/30) | 31.64±1.17  (30/30) | 34.20±1.05  (30/30) | 35.92±0.49  (26/30) | 37.26±1.05  (5/30) | 37.84±1.89  (4/30) |
| HPR primers | Positive | Positive | Positive | Positive | Positive | Positive | Negative | Negative | Negative |
| Seg. 8 primers | Positive | Positive | Positive | Positive | Positive | Negative | Negative | Negative | Negative |

^a^Mean *C*_t_ value (number of replicates with *C*_t_ value/total number of replicates). Each ISAV dilution was tested in 5 replicates which were repeated 6 times for a total of 30 replicates. Thus, using total RNA extracted from cell culture-grown virus, the Snow *et al.* [51] probe and conventional RT-PCR with HPR primers [9] are 10-fold more sensitive than conventional RT-PCR with segment 8 primers [54].
