# Supplementary material for: Discovery of variant infectious salmon anaemia virus (ISAV) of European genotype in British Columbia, Canada
Source: Virol J. 2016 Jan 6;13:3. doi: 10.1186/s12985-015-0459-1 (PMC4702313; doi:10.1186/s12985-015-0459-1)
Supplement: Additional file 2: — Fish tissue samples testing “non-negative” for infectious salmon anaemia virus (ISAV) from 2012-2013 1 . Table listing the ISAV segment 8 tests done in replicates, which were in some cases repeated. Values in this column represent how many replicates produced a C t value, or the averaged result of tests. (DOC 117 kb) [file 12985_2015_459_MOESM2_ESM.doc]

**Supplementary Table S2: Fish tissue samples testing “non-negative” for infectious salmon anaemia virus (ISAV) from 2012-2013^1^. The ISAV seg 8 tests were done in replicates, which were in some cases repeated. Values in this column represent how many replicates produced a *C*_t_ value, or the averaged result of tests.**

| Lab # | Fish# | Species | Internal control, Atlantic salmon EF1α Probe, Cts | Fish Origin | ISAV seg 8 probe detects all ISAV | Conventional RT-PCR Seg 8 Devold primers | Conventional RT-PCR Seg 6 HPR primers | Pooled tissue |
| --- | --- | --- | --- | --- | --- | --- | --- | --- |
| VT10142011-60 | 2 | Chinook | not done | freshly dead | 32.99 | not done | not done | Gill, Heart |
| VT10142011-67 | 10 | chum | not done | freshly dead | 33.77(1/2) | not done | not done | Gill, Heart |
| VT10142011-58 | 11 | coho | not done | frozen | 33.61(1/2) | not done | not done | Gill, Heart |
| VT06172013-81 | 14-Jun | sea louse | not done | freshly dead | 34.08(1/2) | Negative | Negative | Entire Body |
| VT06202012-387 | BA01 | herring | 25.99 | freshly dead | 0 | Positive (European) | Negative | Gill, Heart |
| VT06202012-388 | BA02 | pink | 23.73 | freshly dead | 0 | Positive (European) | Negative | Gill, Heart |
| VT08092012-419 | BB01 | sockeye | 19.55±0.01 | freshly dead | 0 | Positive (European) | Positive (HPR5) | Gill, Heart |
| VT08092012-420 | BB02 | sockeye | 19.07±0.01 | freshly dead | 0 | Positive (European) | Negative | Gill, Heart |
| VT08092012-421 | BB03 | Chinook | 21.18±0.29 | freshly dead | 0 | Positive (European) | Negative | Gill, Heart |
| VT08092012-423 | BB05 | sockeye | 19.51± 00 | freshly dead | 0 | Positive (European) | Negative | Gill, Heart |
| VT08092012-425 | BB07 | sockeye | 20.05±0.13 | freshly dead | 0 | Positive (European) | Negative | Gill, Heart |
| VT08092012-431 | BB13 | sockeye | 19.11±00 | freshly dead | 0 | Positive (European) | Positive (HPR5) | Gill, Heart |
| VT08092012-433 | BB15 | sockeye | 18.90±0.04 | freshly dead | 0 | Positive (European) | Negative | Gill, Heart |
| VT08092012-434 | BB16 | sockeye | 17.61±0.13 | freshly dead | 0 | Positive (European) | Negative | Gill, Heart |
| VT08092012-451 | CA01 | Pacific Chub mackerel | not done | freshly dead | 0 | Positive (European) | Negative | Gill, Heart |
| VT05252012-333 | CL003 | cutthroat trout | 23.52 | freshly dead | 0 | Positive (sequenced) | Positive (HPR5) | Gill, Heart |
| VT05252012-334 | CL004 | cutthroat trout | 24.85 | freshly dead | 0 | Positive (European) | Positive (HPR5) | Gill, Heart |
| VT05252012-335 | CL005 | cutthroat trout | 19.58 | freshly dead | 0 | Positive (European) | Positive (HPR5) | Gill, Heart |
| VT05252012-336 | CL006 | cutthroat trout | 22.31 | freshly dead | 0 | Positive (European) | Positive (HPR5) | Gill, Heart |
| VT05252012-337 | CL007 | cutthroat trout | 21.73 | freshly dead | 0 | Positive (not sequenced) | Positive (HPR5) | Gill, Heart |
| VT05252012-338 | CL008 | cutthroat trout | 24.84 | freshly dead | 0 | Positive (European) | Positive (HPR5) | Gill, Heart |
| VT05252012-339 | CL009 | cutthroat trout | 23.54 | freshly dead | 0 | Positive (not sequenced) | Positive (HPR5) | Gill, Heart |
| VT05252012-340 | CL010 | cutthroat trout | 28.06 | freshly dead | 0 | Positive (not sequenced) | Positive (HPR5) | Gill, Heart |
| VT05252012-341 | CL011 | cutthroat trout | 22.5 | freshly dead | 0 | Positive (European) | Positive (HPR5) | Gill, Heart |
| VT05252012-342 | CL012 | cutthroat trout | 22.25 | freshly dead | 0 | Positive (not sequenced) | Positive (HPR5) | Gill, Heart |
| VT05252012-343 | CL013 | cutthroat trout | 25.9 | freshly dead | 0 | Positive (European) | Positive (HPR5) | Gill, Heart |
| VT05252012-344 | CL014 | cutthroat trout | 21.82 | freshly dead | 0 | weak positive (not sequenced) | Positive (HPR5) | Gill, Heart |
| VT05252012-345 | CL015 | cutthroat trout | 20.42 | freshly dead | 0 | Positive (European) | Positive (HPR5) | Gill, Heart |
| VT06202012-391 | CM03 | Chinook | 18.91 | freshly dead | 0 | Positive (European) | Negative | Gill, Heart |
| VT08092012-446 | CP02 | coho | 20.23±0.04 | freshly dead | 0 | Positive (European) | Negative | Gill, Heart |
| VT08092012-447 | CP03 | coho | 20.50±0.02 | freshly dead | 0 | Positive (European) | Negative | Gill, Heart |
| VT08092012-449 | CP05 | coho | 20.26±0.10 | freshly dead | 0 | Positive (European) | Positive (HPR5) | Gill, Heart |
| VT08092012-450 | CP06 | coho | 21.00±0.10 | freshly dead | 0 | Positive (European) | Negative | Gill, Heart |
| VT04272012-290 | LaP1 | Chinook | 28.92 | freshly dead | 30.67±0.28; 30.99±0.16; 0 | Negative | not done | Gill, Heart |
| VT08092012-476 | LL02 | steelhead farmed | 18.22±0.14 | market | 0 | Positive (European) | Negative | Gill, Kidney |
| VT08092012-478 | LL04 | steelhead farmed | 18.93±0.03 | market | 0 | Positive (European) | Negative | Gill, Kidney |
| VT12192012-1058 | LL21 | kokanee | 24.25±0.0 | freshly dead | 37.71(1/2); 0.0 | not done | not done | Gill, Heart |
| VT06032013-57 | LL61 | steelhead farmed | 24.08±0.06 | market | 33.47±0.20; 0 | Negative | Negative | Gill, Kidney |
| VT06032013-58 | LL62 | steelhead farmed | 22.92±0.03 | market | 34.12±0.03; 0 | Negative | Negative | Gill, Kidney |
| VT06032013-60 | LL64 | steelhead farmed | 23.23±0.02 | market | 0 | Positive (European) | Negative | Gill, Kidney |
| VT08092012-481 | MC01 | coho | 20.55±0.02 | freshly dead | 0 | Positive (European) | Negative | Gill, Heart |
| VT08262013-350 | MQ06 | pink | 19.77±0.15 | freshly dead | 0 | Negative | Positive, HPR5 | Gill, Heart |
| VT08092012-406 | NS102 | coho | 22.32±0.01 | freshly dead | 0 | Positive (European) | Negative | Gill, Heart |
| VT08092012-465 | OK03 | chum | 19.92±0.05 | freshly dead | 0 | Positive (European) | Negative | Gill, Heart |
| VT08092012-466 | OK04 | pink | 20.00±0.23 | freshly dead | 0 | Positive (European) | Negative | Gill, Heart |
| VT08092012-468 | OK06 | chum | 19.33±0.03 | freshly dead | 0 | Positive (European) | Negative | Gill, Heart |
| VT08092012-402 | P111 | pink | 20.97±0.07 | freshly dead | 0 | Positive (European) | Negative | Gill, Heart |
| VT08092012-404 | P113 | pink | 22.09±0.27 | freshly dead | 0 | Negative | Positive (HPR5) | Gill, Heart |
| VT12212012-1068 | SK20 | Atlantic | 25.76±0.24 | market | 36.83±0.28; 34.36(1/2); 0.0 | Negative | Positive (HPR0) | Gill, Kidney |
| VT06202012-379 | SS103 | Atlantic | 24.06 | market | 0 | Positive (European) | Negative | Gill, Kidney |
| VT06202012-380 | SS104 | Atlantic | 23.93 | market | 0 | Positive (European) | Negative | Gill, Kidney |
| VT02132013-14 | SS132 | Atlantic | 26.32 ± 0.13 | market | 0 | Negative | Positive (HPR5) | Gill |
| VT03202012-215 | SS32 | Atlantic | 27.94 | market | 0 | Positive (sequenced) | Positive (HPR5) | Gill |
| VT03202012-216 | SS33 | Atlantic | 27.43 | market | 0 | Positive (sequenced) | Positive (HPR5) | Gill |
| VT03202012-217 | SS34 | Atlantic | 26.86 | market | 0 | Positive (sequenced) | Positive (HPR5) | Gill |
| VT03202012-218 | SS35 | Atlantic | 25.98 | market | 0 | Positive (sequenced) | Positive (HPR5) | Gill |
| VT03202012-219 | SS36 | Atlantic | 28.12 | market | 0 | Positive (sequenced) | Positive (HPR5) | Gill |
| VT07192013-209 | SY21 | sockeye | 19.60±0.01 | freshly dead | 35.13(1/2); 0 | not done | not done | Gill, Heart, Kidney, Spleen |
| VT02142012-52 | TT03 | Atlantic | not done | market | 35.31±0.72 | Negative | Positive (HPR5) | Gill, Kidney |
| VT02142012-65 | TT11 | Atlantic | not done | market | 33.97±0.42 | Negative | Negative | Gill, Kidney |
| VT02142012-87 | TT23 | Atlantic | not done | market | 36.15(1/2) | Negative | Negative | Gill |
| VT02142012-90 | TT25 | Atlantic | not done | market | 35.57(1/2) | Negative | Positive HPR5,HPR7b | Gill, Kidney |
| VT02142012-91 | TT26 | Atlantic | not done | market | 34.65(1/2) | Negative | Negative | Gill |
| VT03202012-190 | TT38 | Atlantic | 28.63 | market | 0 | Not done | Positive (not sequenced) | Gill, Kidney |
| VT03202012-193 | TT41 | Atlantic | 24.97 | market | 0 | Positive (sequenced) | Negative | Gill, Kidney |
| VT03202012-194 | TT42 | Atlantic | 25.91 | market | 0 | Positive (sequenced) | Negative | Gill, Kidney |
| VT03202012-195 | TT43 | Atlantic | 25.55 | market | 0 | Positive (sequenced) | Negative | Gill, Kidney |
| VT03202012-196 | TT44 | Atlantic | 27.51 | market | 0 | Positive (sequenced) | Negative | Gill, Kidney |
| VT03202012-199 | TT47 | Atlantic | 25.56 | market | 0 | Positive (sequenced) | Positive (HPR5) | Gill, Kidney |
| VT03202012-200 | TT48 | Atlantic | 27.08 | market | 34.07(1/2) | Positive (sequenced) | Negative | Gill, Kidney |
| VT03202012-201 | TT49 | Atlantic | 27.16 | market | 0 | not done | Positive (not sequenced) | Gill, Kidney |
| VT03202012-202 | TT50 | Atlantic | 26.56 | market | 0 | Positive (sequenced) | Positive (HPR5) | Gill, Kidney |
| VT03202012-203 | TT51 | Atlantic | 27.98 | market | 35.00(1/2) | not done | Positive (HPR5) | Gill, Kidney |
| VT03202012-204 | TT52 | Atlantic | 28.38 | market | 0 | Positive (sequenced) | Negative | Gill, Kidney |
| VT05012012-308 | TT65 | Atlantic | not done | market | 0 | Positive (sequenced) | not done | Gill |
| VT01222013-03 | TT95 | Atlantic | 22.91±0.03 | market | 0 | Positive (sequenced) | Negative | Gill |
| VT02142012-120 | VR5 | chum | not done | freshly dead | 36.98±0.53 | Positive (sequenced) | Positive (HPR5) | Gill, Heart |
| VT10042011-26 |  | sockeye | not done | frozen | 29.82 | not done | not done | Gill |
| VT10042011-36 |  | sockeye | not done | frozen | 30.86 | not done | not done | Gill |

^1^All samples tested for ELF-1α and ISAV by RT-qPCR; cycle threshold (*C*_t_) values obtained are listed. Samples were then tested using conventional RT-PCR targeting segment 8 [54] and segment 6 HPR [9]. All PCR products from the conventional RT-PCR assays were confirmed by DNA sequencing (and genotype or HPR type established). Fresh tissue samples testing positive for ISAV in any of these three assays were subjected to virus isolation attempts.
